# Validation of Adhesive and Temperature Property Characteristics of Microsurfacing by Performance-Based Mixture Design Approach

**DOI:** 10.3390/ma14164532

**Published:** 2021-08-12

**Authors:** Jicun Shi, Shili Jia, Lan Wang, Qing Zhang, Hongxing Han, Yilong Chen, Zige Song, Lei Zhao

**Affiliations:** 1 School of Civil Engineering and Architecture, Xinxiang University, Xinxiang 453003, China; jicun.shi070@xxu.edu.cn (J.S.); jiahan911@163.com (S.J.); wanglan051112@163.com (L.W.); hxhan@whu.edu.cn (H.H.); c18237307833@163.com (Y.C.); soloszg@gmail.com (Z.S.); 2 National Engineering Laboratory for Highway Maintenance Equipment, Xinxiang 453003, China; 3 Collaborative Innovation Center of Henan Province for Fine Chemicals Green Manufacturing, Key Laboratory of Green Chemical Media and Reactions, Ministry of Education, School of Chemistry and Chemical Engineering, Henan Normal University, Xinxiang 453007, China

**Keywords:** microsurfacing, performance-based, mixture design, response surface method, comprehensive properties

## Abstract

Performance-based mixture design of microsurfacing offers a promising solution to the best application of asphalt emulsions. The presented study investigated a novel approach to evaluate the spalling resistance and high and low-temperature resistance of microsurfacing. The laboratory tests, including mixture bond strength (MBS), driving wheel pavement analyzer (DWPA), multi-stress creep recovery (MSCR), load wheel rutting (LWR), and single edge notch beam (SENB) were conducted to characterize the performance-related properties; the response surface method (RSM) was used to obtain the optimal proportions of the mixture. According to the experimental results, the performance-based mixture design method improves the comprehensive performance of microsurfacing, such as adhesion at high and low temperatures. The results of RSM show that temperature is the most important factor that affects the adhesion of mixture. There is a strong correlation between adhesive and temperature performance detected by different test methods. Due to different chemical mechanisms caused by cement and emulsified asphalt, the high-temperature performance index of the microsurfacing mixture is lower than that of HMA. Furthermore, the low-temperature resistance is analyzed and suggested indicator is proposed.

## 1. Introduction

Microsurfacing is widely used in pavement preventive maintenance, which lays a mixture of aggregate, polymer-modified asphalt emulsion, cement, water, and additives to the right road at the right time to improve the pavement condition of waterproof and skid resistance [1,2]. Microsurfacing can be mixed and spread on-site without heating, and its strength will form after the breaking of the emulsified asphalt [3]. Mixture design and performances of microsurfacing are evaluated using specifications designated by the International Slurry Surfacing Association (ISSA) [4]. Moreover, microsurfacing is considered a sustainable technology due to its construction speed and being environmentally friendly [5]. Despite the attractive merits of microsurfacing, there are some inherent problems that limit its quality and then affect its durability. The control performance indicators of emulsified asphalt used for pavement maintenance are antiquated test approaches when compared to modern asphalt laboratory methods related to filed performance, such as spalling resistance, high-temperature resistance, crack resistance, and traffic [6]. One potential solution is to design the microsurfacing mixture based on its performance.

Since the early 2000s, the performance-based emulsified asphalt specifications have attracted considerable attention [7]. The traditional test method of evaporating the residue at 163 °C is not suitable for the evaluation of modified asphalt emulsion to improve the performance of binder because latex and polymer in asphalt emulsion will age at a high temperature, and it is not consistent with its field application scenarios [8,9]. At the same time, the rheological test of evaporation residue at 60 °C was developed to evaluate the performance of asphalt [10]. The viscosity, shrinkage cracks reveling, fatigue reveling, and blending of evaporation residue were studied. Frequency sweep and multiple stress creep and recovery (MSCR) tests were conducted to evaluate the bleeding and rutting resistance at high temperature [11,12], Bending beam rheometer (BBR) tests were used to predict properties at low temperature [13,14,15].

Because the microsurfacing mixture is composed of emulsified asphalt and cement, its reaction mechanism is different from the hot mixture asphalt (HMA). The properties of emulsified asphalt evaporation residue cannot evaluate the comprehensive performance of microsurfacing [16,17]. The conventional mixture design of microsurfacing used wet track abrasion test (WTAT), load wheel test (LWT), and stability and resistance to conduct compaction tests. In order to adapt to the field application, the Model Mobile Load Simulator (MMLS 3) was developed to evaluate the rutting and bleeding, and the SENB test was developed for temperature cracking [18,19]. Many studies have been carried out on the influence of the composition of emulsion, fiber, cement and others, as well as the molding method, curing time, and temperature performance of microsurfacing mixture [20,21]. Meanwhile, the spalling resistance of microsurfacing was a result of the chemical interaction between the emulsion, cement and aggregate [22,23].

Although the performance of microsurfacing mixtures has been investigated by many studies, comprehensive research including adhesive, rutting, and cracking resistance using performance-related mixture design methodology is still limited. To this end, this study aims to validate the overall performance-related microsurfacing mixtures by characterizing the mixtures’ bond strength and mass loss rate at dry and wet condition, rutting resistance, and cracking resistance. It is expected to enhance the field performance of microsurfacing by performance-related mixture design.

## 2. Materials and Sample Preparation

### 2.1. Materials

The emulation asphalt used in this study was cationic quick setting emulsion supplied by Wisend Road Materials Ltd., Henan, China. MS-A was emulsified SBS-modified asphalt; MS-B and MS-C were both SBR-modified emulsified asphalt. Table 1 shows the basic properties of the three types emulation asphalt used in this study.

The mineral aggregate was basalt stone, whose mechanics and sand was equivalent to the technical requirements of a road surface layer. Table 2 shows the mineral aggregate gradation of microsurfacing.

### 2.2. Sample Preparation

Microsurfacing consists of a mixture of aggregate, emulsified asphalt, mineral powder, cement, water, latex, and additives [24]. Different from asphalt concrete or cement concrete, cement-emulsified asphalt mortar is different from cement paste or asphalt mortar in its concrete strength formation mechanism, structure, and road performance [25,26]. In this paper, the mixture composed of fine aggregate was studied. First, aggregate 200 g, cement 4 g, and sufficient water (about 8%) were well mixed. Then, 6.3% pure asphalt ± 1% emulsified asphalt were added into the mixture. Last, the slurry mixture was poured into the mold coated with an isolating agent on the inside wall; follow-up test was to be carried out after curing.

## 3. Testing Program

**Figure 1:**
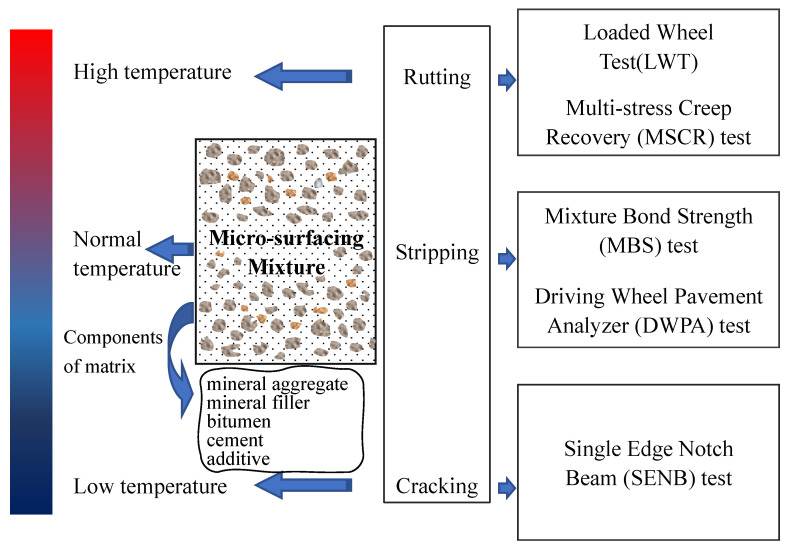
Experimental scheme.

The performance properties of microsurfacing mixtures were verified by laboratory tests. To appraise the adhesive property characteristics of microsurfacing mixtures, mixture bond strength (MBS) test, and driving wheel pavement analyzer (DWPA) test were conducted at a temperature 25 °C. The adhesive property was evaluated by the bond strength and weight-loss rate of the microsurfacing mixtures. The temperature properties of microsurfacing mixtures contain rutting resistance and thermal cracking resistance [13,27]. The resistance to perpetual rutting of the mixture was determined by loaded wheel test (LWT) [28]. The high temperature stability of evaporation residue was examined using MSCR test [29]. Thermal cracking at low temperature was conducted via an SENB test. The experimental scheme of the mixture characterization is shown in Figure 1.

### 3.1. Mixture Bond Strength (MBS) Test

**Figure 2:**
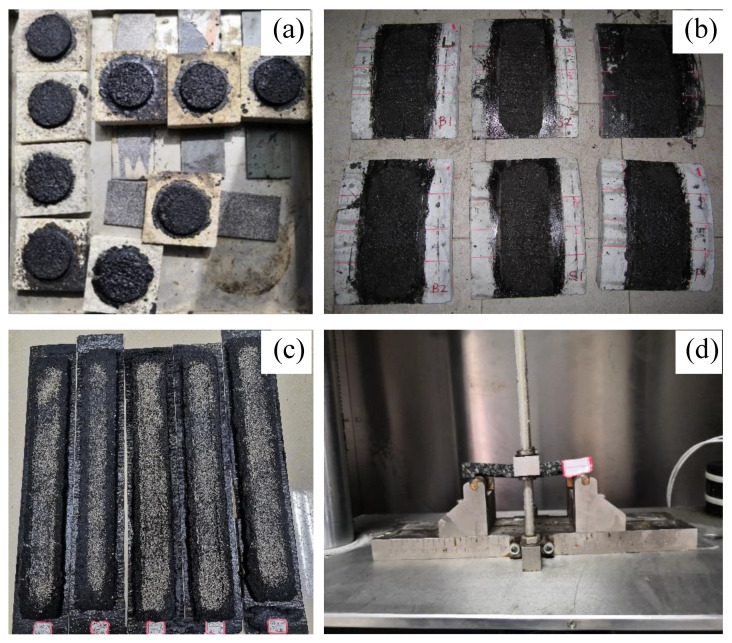
Conducted tests: (**a**) MBS, (**b**) DWPA, (**c**) LWT, and (**d**) SENB.

A 50 mm diameter mold was used to study the bond strength of the microsurfacing mixture to the stone (Figure 2a). The mold was made from a steel ring with an inner diameter of Φ 50 mm and a thickness of 5 mm.

The specimens prepared was prepared as detailed in Section 2.2, and then placed into an oven at 60 °C ± 3 °C drying to a constant weight, generally not less than 16 h. After cooling to room temperature, the AB adhesive evenly applied to the bonding surface of the specimens to ensure firm adhesion. The MBS test was carried out at a temperature of 31 °C. When testing the adhesion properties in a wet state, the specimens need to be immersed in 25 °C water for 24 h. During the test, if the mixture was not adequately formed, most of the adhesive distress that occurred was within the mixture and not at the bonding interface between the specimens and the stone. To fully reflect the bonding performance of the mix and stone at the microsurfacing, on the one hand, the surface finish of the stone was improved to ensure a smooth surface, on the other hand, the regeneration time in the oven was extended to 24 h.

To improve the efficiency of the stone substrate used for the test, the stone slab after drawing was cleaned of residual mixture on the surface of stone with a spatula and the asphalt residue was wiped away with clean cotton yarn dipped in petrol until the surface of stone was clean and dried in an oven at 105 °C for the next test. In order to improve the efficiency of stone substrate, the residual mixture on the stone surface was cleaned with a spatula, the asphalt residue was wiped with a clean cotton yarn dipped in gasoline until the stone surface is clean. At last, the mixture was dried in an oven at 105 °C for the follow-up test.

### 3.2. Driving Wheel Pavement Analyzer (DWPA) Test

To assess the spalling resistance of the microsurfacing mixture, the DWPA test was developed to simulate the effects of vehicle loading and temperature. The main accelerated loading systems used to simulate pavement performance were the Hamburg rutting tester, the accelerated loading facility (ALF), RIOH track, and the one-third scale model mobile load simulator (MMLS3) [30]. The above-mentioned accelerated loading devices have loaded tires that were dragged and passively rotated, which were unable to simulate the interaction between the main drive wheel and the road surface; therefore, this type of pavement accelerated loading equipment can only evaluate the load-bearing capacity of the pavement structure, but not the surface functions of the pavement, such as durability of the road surface paving materials, abrasion (de-granulation), reflection cracking, anti-rutting performance, pavement noise, friction coefficient, water damage resistance, etc. It is also impossible to evaluate the use effect of the surface disposal-based pavement preventive maintenance. DWPA was developed by South China University of Technology (SCUT), using pneumatic rubber tires to drive the rotation of the specimens, which can realistically reproduce the sliding friction between the pavement and tires.

The loading wheel was driven by a speed-controlled motor with a tire pressure of 0.7 MPa. The loading wheel drives the rotation of the specimen wheel (large wheel) by friction to realistically simulate the interaction between tire and pavement, which was used to evaluate the performance of the functional pavement material on the road surface. The specimen wheel can be fitted with eight specimens at the same time (Figure 2b). The device worked in a sealed chamber where the temperature and humidity can be controlled.

**Figure 3:**
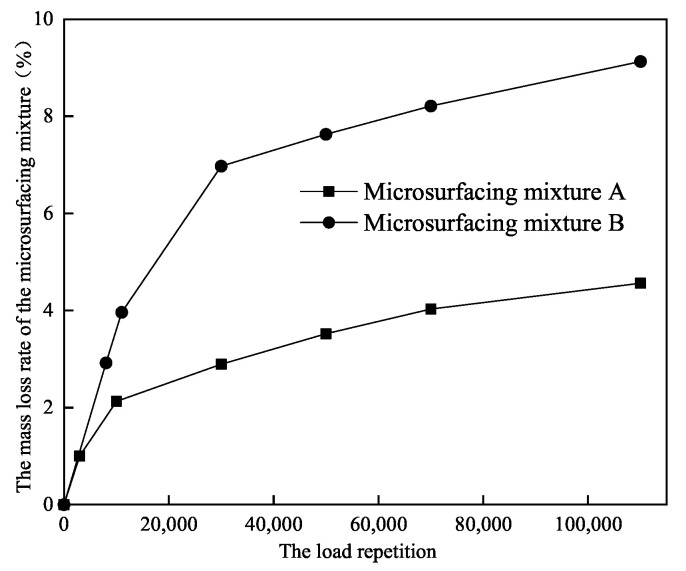
The relationship between mass loss rate and load repetition of microsurfacing mixture.

With the increase of loading times, the adhesion performance decreases continuously due to the fatigue of the test specimens, resulting in the spalling of the pavement under the combined action of tire pressure, friction resistance, and centrifugal force. The spalling resistance was determined by the mass loss of the curved plate specimens after different loadings, the mass loss rate of the microsurfacing mixture under different loadings were calculated. Figure 3 shows a typical relationship between the mass loss rate of a microsurfacing mixture and the number of loadings.

### 3.3. Rutting Resistance and Surfacing Bleeding

#### 3.3.1. Multi-Stress Creep Recovery (MSCR) Test

The multi-stress creep recovery (MSCR) test was proposed by the United States Federal Highway Administration to replace the AASHTO M 320-05 high-temperature specification parameter and various SHRP test methods [31]. The new parameter of non-recoverable compliance, J_*nr*_, was currently being considered as a replacement for the Superpave high-temperature binder parameter of |G∗|/sinδ(ω=10 rad/s) (AASHTO TP 70-11) [32].

The MSCR test was a good selection to evaluate the high-temperature performance of emulsified asphalt residues because it allows tests at different stress levels and temperatures. Studies have shown that binders with low irrecoverable creep flexibility were more resistant to deformation, slippage, and bleeding than those with high irrecoverable creep flexibility. It had also been shown that binders with high elastic recovery properties were also more resistant to rutting, due to their ability to recover to the original deformation. King carried out MSCR tests on a large number of modified and unmodified emulsified asphalts and the results showed that the MSCR tests were able to distinguish modified emulsified asphalt from common emulsified asphalt [8]. Hanz also evaluated the effectiveness of emulsified asphalt using MSCR when performing a gravel seal in Wisconsin, and the MSCR test was able to distinguish between different emulsified asphalt types and the effectiveness of the modification [18]. In this section, in order to evaluate the high-temperature performance, MSCR tests were carried out on emulsified asphalt after steaming the residue at low temperature.

#### 3.3.2. Loaded Wheel Test (LWT)

The wheel rutting deformation test was used to evaluate the rutting resistance of microsurfacing mixtures. In this study, the LWT was used to test the width deflection rate PLD of the specimens according to the test method of the microsurfacing and slurry seal mixes guide, which was calculated from Equation (1).

(1) $$PLD=\frac{(L_{b}-L_{a})\times 100}{L_{a}}$$

where $L_{b}$—width of rolled specimens (mm), $L_{a}$—width of specimens before rolling (mm), PLD—deformation rate per unit width (%).

### 3.4. Low-Temperature Resistance Test

The cracking resistance of microsurfacing mixture at low temperature is an important factor affecting the durability [33]. In this research, the anti-cracking performance of the single edge notch beam (SENB) test proposed by Wagoner was studied. The size of specimen was 6.25 mm × 12.5 mm × 102 mm, and notch size was 3 mm. The rotary compactor was used for controlling the target void ratio to 10%, and the specimen with a diameter of 150 mm and a height of 67 mm was formed. Then, several SENB specimens were cut, and the UTM tester was used to the bending test. The temperature of UTM environmental chamber was −10 °C, the distance between two points of the test frame (loading mid-span distance) was 101.6 mm (80% of the length of the specimen), and the load was applied at 0.6 cm/min until the specimen damaged [34]. The failure load and displacement at the time of failure was recorded. Figure 2d shows the beam bending test.

### 3.5. Optimum Material Parameters and Experimental Design Using RSM

The first step in preparing the specimens was to determine the dosages of cement and emulsified asphalt. Based on empirical data, the dosages of emulsified asphalt were chosen to be 5.8%, 6.5%, and 7.0%, respectively. The dosages of cement were initially proposed to be 1%, 1.5%, and 2%. The amount of external water was adjusted according to the temperature and type of aggregate through the mixable time, generally from 3% to 7%. The samples were cured in a blast drying oven at 25 °C, 40 °C, and 60 °C for not less than 40 h. The type of emulsified asphalt used for the experiments was MS-A. The Box–Behnken design (BBD) RSM was used for the experimental design. RSM is a combined mathematical and statistical method that optimizes response values by modeling and analyzing multiple variables affecting response values. The optimal combination of multiple variables was obtained by a deterministic test, using the polynomial function to fit the functional relationship between factors and response values, establishing regression models, and optimizing process parameters [35]. Experimental design, mathematical modeling, prediction of response values, and verification of the accuracy of the model are the three main components of the response surface approach to process optimization. In contrast to orthogonal experiments, it is possible to derive the interaction between multiple factors in a limited number of experiments and the regression equation is highly accurate. Near the best point of the response surface, the curvature effect is the dominant term, and the response surface is approximated by the second-order model. The response value was calculated by Equation (2).

(2) $$Y=\beta_{0}+\sum_{i=1}^{k}\beta_{i}X_{i}+\sum_{i=1}^{k}\beta_{ij}X_{i}^{2}+\sum_{i<j}^{k}\beta_{ij}X_{i}X_{j}+\varepsilon$$

where *Y*—response value, $X_{i},X_{j}$—the *i*th and *j*th experimental factors, β—regression coefficient, *k*— the number of test, ε— random error.

The BBD design table was based on the second-order model design with a central point, and the experimental table was arranged in code form as shown in Table 3. The number of experiments in this study was based on the BBD code with 15 experimental points, twelve of which were analyzing points and three were zero points to estimate the error. Using the experimental matrix designed in Table 3, a total of 180 specimens including bonding, accelerated loading, wheel rutting deformation and SENB were produced and three parallel experiments were conducted for each experiment. Table 3 shows the experimental results.

The parameters in Table 3, X_1_—the dosage of emulsified asphalt (in terms of bitumen to stone ratio) (%), X_2_—amount of cement (%), X_3_—test temperature (°C), MBS_*dry*_, MBS_*wet*_—bond strength in dry and wet state (MPa), DWPA_*dry*_, DWPA_*wet*_—rate of mass loss after 10,000 accelerated loads in dry and wet state (%), PLD—deformation rate per unit width (%), SENB—maximum displacement determined by single edge notch beam (SENB) test (mm).

## 4. Results and Discussion

### 4.1. Optimum Comprehensive Properties Determination

**Figure 4:**
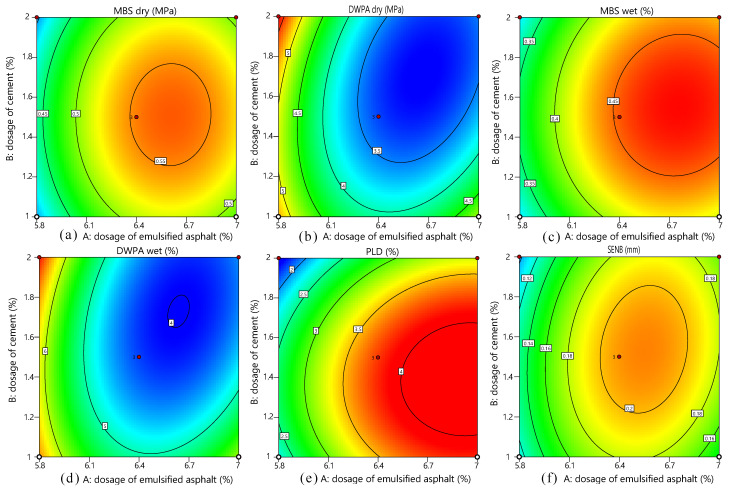
Contour map of the optimized experimental results: (**a**) ${\mathrm{MBS}}_{\mathrm{dry}}$, (**b**) ${\mathrm{MBS}}_{\mathrm{wet}}$, (**c**) ${\mathrm{DWPA}}_{\mathrm{dry}}$, (**d**) ${\mathrm{DWPA}}_{\mathrm{wet}}$, (**e**) PLD, and (**f**) SENB.

The dosage of emulsified asphalt has a greater impact on the performance of the mixture high temperature, low temperature, and anti-spalling properties. In addition, the higher the curing temperature, the faster the molding speed, meaning that the mixture shows higher bond strength and low and high-temperature performance. The increase in the amount of cement can significantly enhance the high-temperature resistance of the mixture, in order to evaluate the impact of different test parameters on the performance of mixture. The RSM was used to the experimental design and results analysis. The contour plots of different experimental parameters are shown in Figure 4. We can see that all indicators have changed significantly when the amount of emulsified asphalt (binder to stone ratio) changed from 6.4% to 6.6% and the amount of cement changed from 1.4% to 1.6%. In addition, as the temperature of curing increases from 25 °C to 60 °C, the evaporation rate of water in the mixture at the microsurfacing is accelerated, which is beneficial to its forming. So the temperature of 60 °C is the best curing temperature, and 60 °C the maintenance will not cause aging. At the same time, it will maintain a certain amount of moisture to facilitate the hydration of the cement in the mixture and improve the strength of the mixture.

Statistical analysis of the experimental data was carried out by means of the RSM, and the optimum experimental conditions and results were optimized. From the experimental objectives, the maximum value of MBS for bond strength is expected to be obtained in both dry and wet conditions. The minimum value of aggregate loss rate is expected to be obtained for accelerated loading tests. The minimum rate of wheel rutting deformation, which reflects the high-temperature resistance of the mix. The maximum displacement of the single-sided notched beam, which reflects the low-temperature resistance. Based on the above objectives, an ANOVA of the test data was carried out, resulting in the following response surface Equations (3)–(8) for the regression models of the different influencing factors.

**Figure 5:**
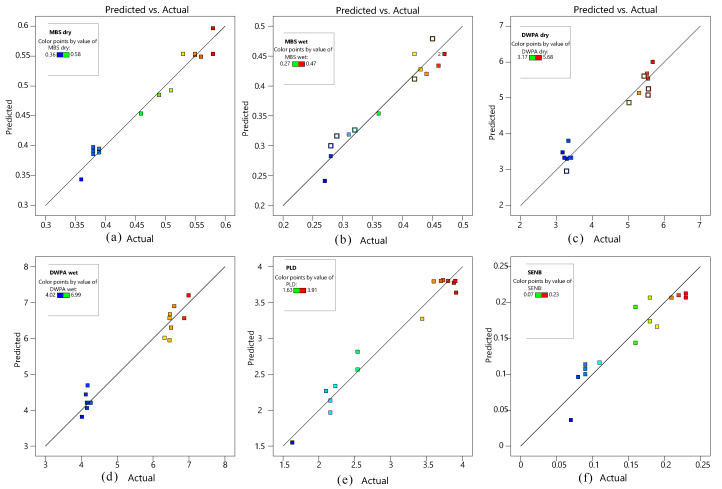
The linear simulation of predicted versus actual values: (**a**) ${\mathrm{MBS}}_{\mathrm{dry}}$, (**b**) ${\mathrm{MBS}}_{\mathrm{wet}}$, (**c**) ${\mathrm{DWPA}}_{\mathrm{dry}}$, (**d**) ${\mathrm{DWPA}}_{\mathrm{wet}}$, (**e**) PLD, and (**f**) SENB.

The ANOVA results can be seen Table 4; the three factors of emulsified asphalt dosage, cement dosage, and conditioning temperature have significant effects on the test results, which in turn indicate that the response surface model can be used to design reasonable parameters to produce the desired test results. The statistical significance of the experimental results are shown in Figure 5.

(3) $$\begin{array}{cc} MBS_{dry} & =-10.87+3.39X_{1}+0.52X_{2}-0.27X_{1}^{2}-0.17X_{2}^{2} \end{array}$$

(4) $$\begin{array}{cc} MBS_{wet} & =-8.19+2.41X_{1}+0.48X_{2}+0.013X_{3}-0.19X_{1}^{2}-0.147X_{2}^{2} \end{array}$$

(5) $$\begin{array}{cc} DWPA_{dry} & =149.59-46.60X_{1}+5.05X_{2}+0.11X_{3}-1.81X_{1}X_{2}-0.05X_{1}X_{3}+3.98X_{1}^{2}+1.95X_{2}^{2}+1.95X_{3}^{2} \end{array}$$

(6) $$\begin{array}{cc} DWPA_{wet} & =187.98-58.46X_{1}+4.89X_{2}+0.144X_{3}-1.91X_{1}X_{2}-0.06X_{1}X_{3}+4.94X_{1}^{2}+2.15X_{2}^{2} \end{array}$$

(7) $$\begin{array}{cc} PLD & =-87.23+23.54X_{1}+9.03X_{2}+0.25*X_{3}-0.045X_{2}X_{3}-1.71X_{1}^{2}-2.59X_{2}^{2} \end{array}$$

(8) $$\begin{array}{cc} SENB & =-0.30+0.05X_{1}+0.005X_{2}+0.003X_{3} \end{array}$$

**Figure 6:**
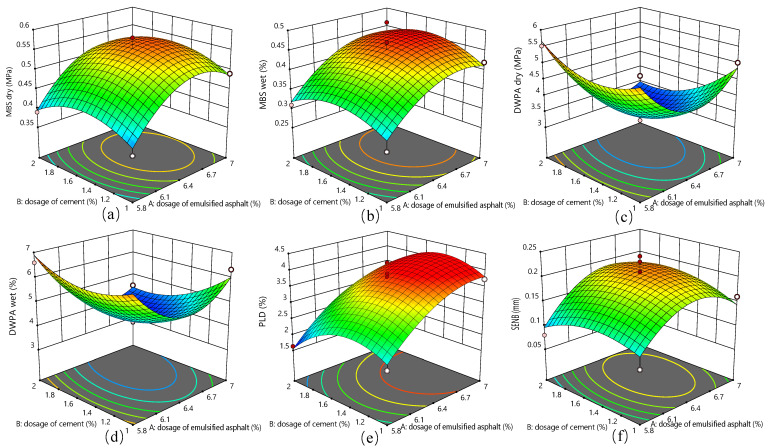
Effect of emulsified asphalt, cement, and temperature on the characteristics: (**a**) ${\mathrm{MBS}}_{\mathrm{dry}}$, (**b**) ${\mathrm{MBS}}_{\mathrm{wet}}$, (**c**) ${\mathrm{DWPA}}_{\mathrm{dry}}$, (**d**) ${\mathrm{DWPA}}_{\mathrm{wet}}$, (**e**) PLD, and (**f**) SENB.

The three-dimensional contour clouds of the response surfaces of the three factors are shown in Figure 6. The effect of each factor on the test results can be visualized in the figure, which shows that an emulsified asphalt dosage of 6.5%, a cement dosage of 1.5%, and a conditioning temperature of 60 °C result in the optimum spalling and high and low-temperature resistance of the mixture. The results of RSM optimization are basically consistent with the results verified by experiments, which shows that the experimental design using RSM is in line with the expected results.

Table 5 shows that an emulsified asphalt dosage of 6.5%, a cement dosage of 1.5% and the curing temperature at 60 °C result in the optimum spalling and high and low-temperature resistance of the mixture. The results of the response surface method optimization are in general agreement with those obtained by experimental verification, which meets the expected results.

**Figure 7:**
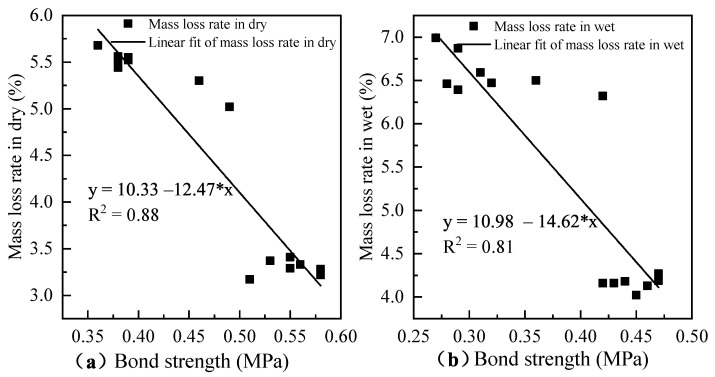
The relationship between mass loss rate and bond strength of mixture: (**a**) masss loss rate in dry, and (**b**) mass loss rate in wet.

To investigate the relationship between the bond strength and accelerated loading, the mass loss rate and the bond strength after accelerated loading were further regressed and the results are shown in Figure 7. It can be seen from the figure, with the increase of bond strength, the mass loss rate of the specimens after accelerated loading gradually decreased. There is a good correlation between the bond strength and mass loss rate of the specimens after drying and immersion, with correlation coefficients $R^{2}$ of 0.87 and 0.79, respectively. The bond strength of the specimens after immersion loses about 11% to 40% compared to that of the dry condition. The mass loss rate of the specimens after immersion is larger than that of the dry state. The slope of the loss rate is larger in absolute value than that of the dry condition, indicating that water has a significant damaging effect on their properties.

**Figure 8:**
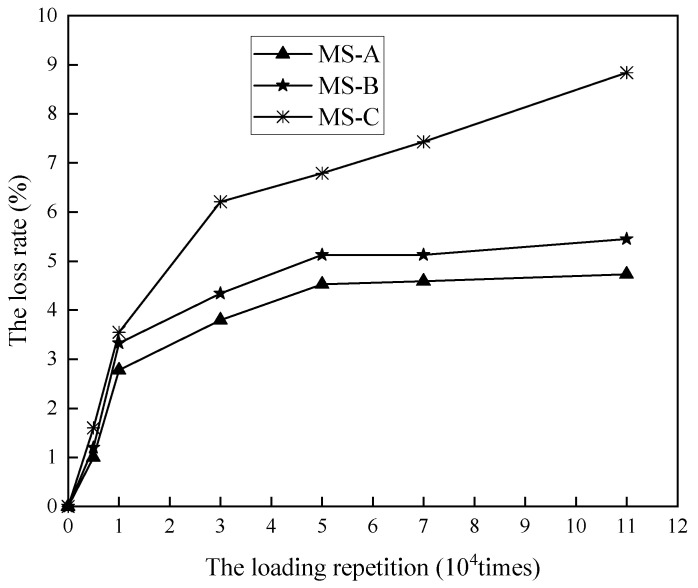
The relationship between mass loss rate and loading repetition.

To further analyze the spalling resistance of the mixture at the microsurface, accelerated loading specimens were made using three types of MS-A, MS-B, and MS-C to analyze the mass loss rate at different loading times, the results of which are shown in Figure 8. Of these, the microsurfacing paving with MS-C in the field was known to have poor spalling resistance. The accelerated loading test results show that the mass loss rate increases rapidly with the number of loadings, then grows slowly and then stabilizes. This is due to the microscopic redistribution of stone and minerals at the microsurface of the mixture under load, which in turn affects the spalling performance of the mixture. The inflection point in the mass loss rate generally occurs at 10,000 loading times and stabilizes at 50,000 times.

It can be seen from Figure 8 that the wear loss increases sharply before the loading times reach 30,000 times. There is no obvious difference in mass loss rate of different types of emulsified asphalt specimens under 5000–10,000 loads. Conventional wet wheel abrasion tests do not take into account the effect of the number of load actions on the performance of the specimens. As such, it is not possible to distinguish between the effects of load, especially for heavy-traffic sections where the emulsified asphalt chosen is significantly different from the emulsified asphalt and other materials for light-traffic sections.

### 4.2. High-Temperature Deformation Resistance Results and Analysis

**Figure 9:**
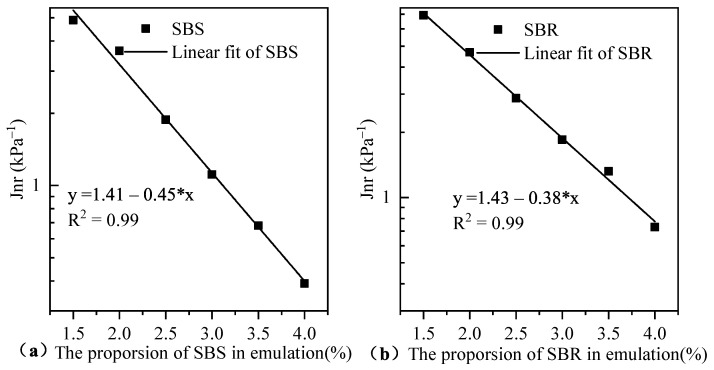
Variation of $J_{nr}$ with the content of SBS/SBR in emulsified asphalt.

The MSCR tests are based on a stress level of 64 °C and 3.2 kPa (Figure 9 for test results), the standard MSCR procedure has two stress levels of 0.1 kPa and 3.2 kPa [36,37]. The unrecoverable creep flexibility ($J_{nr}$) at 3.2 kPa is also used to evaluate the resistance of emulsified asphalt residues to high temperature rutting and bleeding levels. The higher $J_{nr}$ indicates a higher susceptibility to rutting and an increased probability of rutting and bleeding [38]. MS-A was emulsified SBS-modified asphalt, while MS-B was SBR, both of which were quick-setting cationic emulsified asphalt, and both contain 3% additives. It was found that the width deflection rates of MS-A and MS-B emulsified asphalt were 3.73 and 4.12, respectively, which met the requirement of not more than 5%. The study was carried out to evaluate the performance of emulsified asphalt with different SBS and SBR dosing levels and to compare the performance with the width deflection rate of the rutting test.

The creep recovery characteristics of the emulsified asphalt evaporative residues were tested using MSCR tests at 64 °C and 3.2 kPa stress level for the regional temperature characteristics, and the double logarithmic coordinate axis of the distribution of $J_{nr}$ values with SBS and SBR admixtures is shown in Figure 9. The $J_{nr}$ of the SBR-modified emulsified asphalt evaporated residue was higher than that of SBS-modified emulsified asphalt evaporated residue; however, with the increase of the doping amount, the $J_{nr}$ of both gradually decreased, indicating that their resistance to high-temperature deformation was improved, while the gap between the two gradually decreased, and the non-reversible creep kneading amount of both was getting closer. There was a good power function relationship between the doping amount of SBS and SBR and $J_{nr}$ 3.2 and the R^2^ is greater than 0.96.

There are currently two methods of classifying modified bitumen using MSCR. One is the AASHTO MP19-2010 modified bitumen high-temperature performance classification. According to $J_{nr}$ 3.2 for the classification of different traffic levels, the corresponding traffic levels are divided into standard (S), heavy (H), extra heavy (V), and extreme traffic (E) four levels. AASHTO MP19-2010 lists the high-temperature performance classification on the MSCR test index requirements, as shown in Table 6. Alternatively, Y. Richard Kim et al., in the NCHRP 837 study, proposed three levels of criteria for low, medium, and heavy-traffic and their corresponding $J_{nr}$ values, based on the annual average daily traffic AADT, as shown in Table 6.

It can be seen from Table 6 that there are no differences when the first method uses the equivalent standard axle times ESALs for the classification of traffic and the second uses annual average daily traffic AADT. The first method requires a lower value for $J_{nr}$ 3.2 compared to the second method, i.e., the required permanent deformation is more demanding. When the second method is evaluated, we determined that due to the thin thickness of the microsurfacing, the value of $J_{nr}$ required for medium traffic and high traffic is the same because the aggregates in the mixture basically have no room for migration after 2500 loadings.

**Figure 10:**
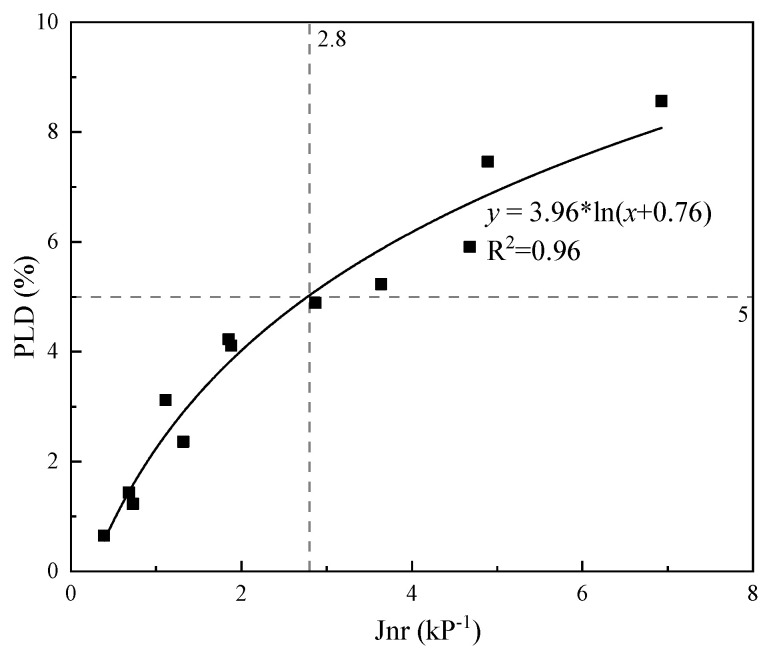
The relationship between $J_{nr}$ and PLD.

Compared with the requirements of $J_{nr}$ 3.2, the modified bitumen is lower than the emulsified asphalt, which can be seen in Table 6. It is probably due to the the fact that the thickness of the HMA is much higher than the microsurfacing mixture. The mixture of aggregates is prone to migration; in order to ensure the high-temperature performance of the mixture, the requirements are more stringent. Secondly, the microsurfacing mixture has doped cement and other fillers, and the cement in the mixture forms a three-dimensional mesh structure, which enhances the permanent deformation resistance of the mixture, so its more widely controlled than that of HMA. Existing specifications, only for the microsurfacing at the mixture filling rutting requirements, the rutting deformation test width change rate is not greater than 5%; however, according to practical experience, the microsurfacing at 1 to 2 years after the opening has a significant decrease in rutting resistance performance. Most of the asphalt was found in the mixture at high temperatures when the migration occurred, resulting in the formation of a dense bleeding on the surface of the microsurfacing. The depth of structure is significantly reduced, which affects its rutting resistance and appearance [39]. Although the microsurfacing mixture is thinner, the requirements for the high-temperature resistance of the emulsified asphalt should not be reduced, especially for microsurfacing overlays. The MSCR should be used to study the performance of emulsified asphalt evaporative residues and to establish the relationship with the high-temperature performance of the mixture. This study further analyzed the relationship between $J_{nr}$ and the rate of change of wheel rutting deflection width and the results are shown in Figure 10.

The larger $J_{nr}$ 3.2, the greater PLD and the poorer resistance to permanent deformation. When the width change rate of rutting deformation test is 5%, the value of $J_{nr}$ 3.2 is 2.8 kPa${}^{-1}$. This result is different from the values specified in the AASHTO MP19-2010 specification and the NCHRP 837 report. The main reason is that the traffic volume calculation standard is different [8,11]. The 5% change in the width of the wheel rutting deformation test studied in this paper should be proposed for sections where heavy traffic has produced ruts. The threshold of emulsified asphalt for microsurfacing mixtures used for heavy and medium traffic should be 2.8 kPa${}^{-1}$. When formulating specific technical indicators, we should combine traffic and material characteristics to develop an indicator system that meets the requirements, rather than simply copying relevant standards or documents.

At low temperatures, the strain or stress inside the asphalt exceeds the ultimate performance of the asphalt and leads to cracking, at which time the strength is the ultimate strength and the corresponding temperature is the ultimate strength temperature. It was evaluated by the BBR results of stiffness and m-value after 60 s loading [40]. The equations are presented in Equations (9) and (10).

(9) $$\begin{array}{cc} S(t) & \approx \frac{3G^{*}\left(\omega \right)}{1+0.2sin(2\delta )} \end{array}$$

(10) $$\begin{array}{cc} m & =\frac{d(logG^{*})}{d(log\omega )} \end{array}$$

where S(t)—creep stiffness at time t, Pa, G∗(ω)—complex modulus at frequency ω, Pa, δ—phage angle at frequency ω, Pa, *m*—slope of G* versus frequency plot at a given frequency.

**Figure 11:**
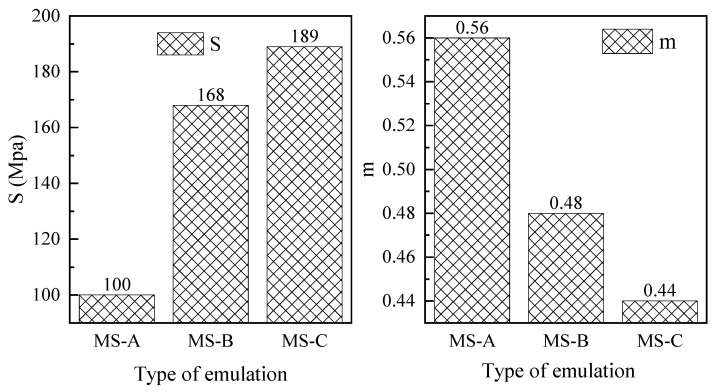
BBR properties from DSR frequency sweep test S(60) and m(60).

The creep relaxation is the change rate of the stiffness over time. If the creep rate is larger, the asphalt relaxation performance is better. On the contrary, if the temperature stress accumulates to a certain degree, thermal cracking will appear. Considering the temperature characteristics of the studied area, DSR frequency scan tests at 0 °C were carried out to derive the BBR creep stiffness modulus S (60 s) and creep relaxation rate m (60 s) values at 60 s, which were used as evaluation indicators. The 60 s creep stiffness modulus S (60 s) test results of three emulsified asphalt at 0 °C are shown in Figure 11. It can be seen from the figure that the creep modulus S (60 s) of MS-A is the lowest and the relaxation rate is the highest, indicating that MS-A emulsified asphalt has the best low-temperature resistance of the three, followed by MS-B, and MS-C has the worst low-temperature resistance.

The performance of microsurfacing mixture is controlled by the adhesive and the high and low-temperature resistance tests. From the test results, if the traditional mixture proportion design method of microsurfacing mixture is adopted, all indexes meet the requirements, but the disadvantages of each material can be clearly seen through the above compatibility test method. For example, the anti-wear performance of MS-C in wet conditions with water can not meet the technical requirements, especially in the original road section with serious water seepage, so there is evidence for large-area spalling after paving.

## 5. Conclusions

This study presents a comprehensive laboratory study to validate the performance of microsurfacing mixture using performance-based mixture design approach. Optimum material parameters were obtained and compared via RSM. Adhesive and temperature resistance of microsurfacing mixture were characterized by MBS, DWPA, LWT, and SENB. Based on the results of this paper, the following major findings are derived:

- When the DWPA test load was performed 10,000 times, the mass loss rate appears at the inflection point, and tends to be stable when the DWPA test load was performed 50,000 times; therefore, the DWPA test is recommended as the test index of bonding performance of microsurfacing mixture.
- Considering adhesive strength, rutting, and cracking resistance, microsurfacing incorporated with 6.5% modified asphalt emulsion, 1.5% cement, and curing at a temperature of 60 °C has optimum comprehensive characteristics. The order of influence on comprehensive performance is asphalt content, temperature, and cement content.
- The $J_{nr}$ value of evaporation residue is an important factor affecting the high temperature stability of mixture, which is slightly lower than the requirement of HMA. The low-temperature cracking resistance of mixture depends on the creep stiffness of evaporation residue.
- The adhesive, rutting, and cracking resistance can be characterized by MBS, DWPA, LWT, and SENB test results. This study proposes to comprehensively utilize those parameters as quantitative evaluation indexes of microsurfacing performance.

Our main findings have confirmed that the designed test method can effectively evaluate the comprehensive performance of the mixture, but the influence of fatigue performance has not been fully verified. It is recommended that additional fatigue performance tests, such as the indirect tensile fatigue test and the four-point beam test, be conducted to further validate the fatigue performance of microsurfacing mixtures.

## Figures and Tables

**Table 1:** Properties of emulsion asphalt.

Type	MS-A	MS-B	MS-C	Required Value
Residual sieve (%)	0.06	0.01	0.01	≤0.01
Standard viscosity $C_{25,3}$	46	32	33	12–60
Residue percentage (%)	61	62	60.5	≥60
Penetration at 25 °C (0.1 mm)	77	78	89	40–150
Softening point (°C)	56	59	56	≥53
Ductility 5 °C	63	67	62	≥20
Solubility	98.5	97.5	99	≥97.5
Storage stability (1 d)	0.2	0.6	0.8	≤1
Storage stability (5 d)	4.8	4.5	2.6	≤5

**Table 2:** Mineral aggravate gradation of microsurfacing.

Gradation Type	Percentage by Mass (%) Passing the Following Sieve (mm)							
	9.5	4.75	2.36	1.18	0.6	0.3	0.15	0.075
MS-3 standard	100	70–90	45–70	28–50	19–34	12–25	7–18	5–15
Actual value	100	80	62.5	39	26.5	18.5	12.5	10

**Table 3:** Result of the experimental design by BBD.

Run	X_1_	X_2_	X_3_	MBS*_dry_*	DWPA*_dry_*	MBS*_wet_*	DWPA*_wet_*	PLD	SENB
1	5.8	1	42.5	0.38	5.56	0.29	6.87	2.1	0.09
2	7.0	1	42.5	0.49	5.02	0.42	6.32	3.73	0.16
3	5.8	2	42.5	0.39	5.52	0.31	6.59	1.63	0.08
4	7.0	2	42.5	0.51	3.17	0.46	4.13	3.44	0.19
5	5.8	1.5	25	0.36	5.68	0.27	6.99	2.16	0.07
6	7.0	1.5	25	0.38	5.44	0.32	6.47	3.88	0.11
7	5.8	1.5	60	0.46	5.3	0.36	6.5	2.23	0.18
8	7.0	1.5	60	0.58	3.28	0.45	4.02	3.6	0.16
9	6.4	1	25	0.39	5.55	0.28	6.46	2.54	0.09
10	6.4	2	25	0.38	5.5	0.29	6.39	2.37	0.1
11	6.4	1	60	0.56	3.33	0.44	4.18	3.91	0.23
12	6.4	2	60	0.55	3.29	0.43	4.16	2.16	0.22
13	6.4	1.5	42.5	0.55	3.41	0.47	4.27	3.7	0.21
14	6.4	1.5	42.5	0.53	3.37	0.42	4.16	3.9	0.23
15	6.4	1.5	42.5	0.58	3.22	0.47	4.19	3.8	0.18

**Table 4:** ANOVA of optimum comprehensive performance designed by BBD.

Response	R${}^{2}$	Adjusted R${}^{2}$	Predicted R^2^	Model F-Value	Model *p*-Value	Significance
$MBS_{dry}$	0.972	0.922	0.727	19.32	0.0023	significant
$MBS_{wet}$	0.936	0.821	0.25	8.14	0.016	significant
$DWPA_{dry}$	0.903	0.773	0.167	6.95	0.0015	significant
$DWPA_{wet}$	0.954	0.871	0.267	11.5	0.0075	significant
PLD	0.956	0.876	0.321	12.03	0.0068	significant
SENB	0.856	0.439	0.264	4.65	0.025	significant

**Table 5:** Comparison of RSM and test results.

No.	*ABS_dry_*	*DWPA_dry_*	*ABS_wet_*	*DWPA_wet_*	*PLD*	*SENB*	Desirability	–
RSM	0.61	2.78	0.48	3.53	3.73	0.2	0.618	Selected
experimental	0.57	2.86	0.46	3.42	3.79	0.21	–	–

**Table 6:** Properties of asphalt emulsion.

Testing Method	Traffic Levels		$J_{\mathrm{nr}}$ 3.2 (kPa^−1^)
	Grade	Traffic Volume	
Modified hot asphalt AASHTO MP19-2010 (no greater than)	Standard(S)	ESALs < 1 × 10${}^{7}$ Standard axle load speed > 70 km/h	4
	Heavy traffic (H)	ESALs 1 × 10${}^{7}$ to 3 × 10${}^{7}$ Standard axle load speed 20∼70 km/h	2
	Overweight traffic (V)	ESALs > 3 × 10${}^{7}$ or standard axle load speed < 20 km/h	1
	Very-heavy traffic (E)	ESALs > 3 × 10${}^{7}$ and standard axle load speed < 20 km/h	0.5
Microsurfacing NCHRP research report 837	Low-traffic volume (L)	AADT < 500	5
	Medium-traffic volume (M)	501 < AADT < 2500	1.5
	High-traffic volume (H)	2501 < AADT < 20,000	1.5

## Data Availability

The data presented in this study are available on request from the corresponding author.
